# Open Pilot Trial of a Coached Digital Program for Lower‐Income Adults With Eating Disorders

**DOI:** 10.1002/eat.24459

**Published:** 2025-05-12

**Authors:** Erin C. Accurso, Catherine R. Drury, Kimberly Yu, Siena Vendlinski, Nancy Jacquelyn Pérez‐Flores, Carli P. Howe, Denise E. Wilfley, Ellen E. Fitzsimmons‐Craft

**Affiliations:** ^1^ Department of Psychiatry and Behavioral Sciences University of California, San Francisco San Francisco California USA; ^2^ Philip R. Lee Institute for Health Policy Studies University of California, San Francisco San Francisco California USA; ^3^ Department of Psychology University of South Florida Tampa Florida USA; ^4^ Department of Psychiatry Washington University in St. Louis School of Medicine St. Louis Missouri USA; ^5^ Department of Psychological & Brain Sciences Washington University in St. Louis St. Louis Missouri USA

**Keywords:** adults, binge eating disorder, bulimia nervosa, eating disorders, health disparities, lower‐income, Medicaid, outcome, socioeconomic status, treatment

## Abstract

**Objective:**

This study evaluated the feasibility, acceptability, and preliminary effectiveness of the first digital intervention tailored for lower‐income adults with eating disorders, who are poorly served by the public health care system.

**Method:**

Adults (*N* = 30) with public insurance or without insurance coverage who endorsed ≥ 6 binge eating episodes, ≥ 6 vomiting episodes, and/or ≥ 6 laxative/diuretic episodes in the past three months with a body mass index ≥ 18.5 kg/m^2^ were enrolled in this open pilot trial. Participants received access to the coached digital CBT‐based intervention, which included individualized guidance and twice‐weekly SMS feedback from a program coach over three months.

**Results:**

Almost all participants (93.3%, *n* = 28) accessed the program after enrollment, completing about half (M = 4.15, SD = 2.68) of the 8 sessions and sending an average of 32.5 (SD = 35.2) texts to their coach over three months. From pre‐ to post‐intervention, there were large improvements in eating disorder psychopathology (*d* = 0.79, *p* < 0.001) and moderate decreases in binge eating (*d* = 0.62, *p* = 0.003) and self‐induced vomiting episodes (*d* = 0.43, *p* = 0.031). There were also large improvements in clinical impairment (*d* = 0.83, *p* < 0.001) and moderate to large reductions in anxiety (*d* = 0.47, *p* = 0.019) and depression (*d* = 0.84, *p* < 0.001). Most participants indicated that they were somewhat to very satisfied with the program (67.9%, *n* = 19).

**Discussion:**

The results from this pilot trial testing a brief online guided self‐help intervention are promising, with relatively high treatment engagement, indicating good feasibility and acceptability and signals of preliminary effectiveness. Future research is needed to examine longer‐term effectiveness relative to other active treatments or waitlist control.

**Summary**

- Lower‐income publicly insured or uninsured adults with eating disorders represent an underserved population at high risk for health disparities.
- This pilot trial demonstrated that lower‐income adults with eating disorders had relatively high rates of engagement and satisfaction with a brief online guided self‐help intervention, with moderate to large changes across clinical outcomes.
- Low‐intensity, lower‐cost treatment may be an effective way of engaging this population in evidence‐based eating disorder treatment.

Eating disorders (EDs) are a significant public health problem (Treasure et al. 2020) with high mortality rates (Ayton et al. 2024; van Hoeken and Hoek 2020) and considerable economic costs (Streatfeild et al. 2021). Despite the prevalent misconception that EDs primarily affect affluent White cisgender women (Sonneville and Lipson 2018), EDs affect individuals from diverse racial, ethnic, and socioeconomic backgrounds (Udo and Grilo 2019; Simone et al. 2022; Huryk et al. 2021). However, less than 20% of those with EDs ever receive treatment (Coffino et al. 2019), with this gap being even more pronounced for those from racial and ethnic minority groups and lower socioeconomic backgrounds (Cachelin et al. 2001; Eisenberg et al. 2011; Grammer et al. 2022; Kazdin et al. 2017).

Individuals with fewer socioeconomic resources are at higher risk for poor outcomes given limited financial resources and other vulnerabilities. They are less likely to receive ED treatment (Sonneville and Lipson 2018) given a number of barriers to accessing care (Penwell et al. 2024), including misdiagnosis and under‐diagnosis (Hart et al. 2011), poor mental health literacy (Grammer et al. 2022; Hamilton et al. 2022; Sonneville and Lipson 2018), feelings of helplessness and denial (Radunz et al. 2023), high treatment costs (Penwell et al. 2024), food insecurity (Frayn et al. 2022), housing insecurity (Bailey‐Straebler et al. 2024), and lack of cultural humility among providers (Heim and Kohrt 2019). Further, publicly funded community‐based healthcare systems typically lack the training, resources, and expertise to identify and treat EDs effectively (Accurso, Buckelew, et al. 2021). Subsequently, poor treatment access contributes to low treatment use, particularly among individuals from under‐resourced communities, leading to gaps in care that may lead to prolonged illness, poorer prognosis, and greater risk of relapse for those with low socioeconomic status, racial and ethnic minorities, and those with Medicaid insurance (i.e., public insurance in the U.S.) or without insurance (Arpey et al. 2017; Cachelin et al. 2001; Forrest et al. 2017; Regan et al. 2017; Vendlinski et al. 2025).

Although clinical guidelines consider cognitive‐behavioral therapy (CBT) the first‐line treatment for bulimia nervosa and binge eating disorder (Hilbert et al. 2017; Waller and Beard 2024), very few clinicians provide this treatment (Kazdin et al. 2017). Indeed, most individuals with EDs who receive treatment do not receive evidence‐based ED treatment (Cooper and Bailey‐Straebler 2015). In the U.S., these interventions are delivered primarily by providers who accept self‐payment only, limiting access to high‐quality care primarily to the few who can pay outofpocket for mental health care (Accurso, Buckelew, et al. 2021; Accurso, Mu, et al. 2021). And even among those who specialize in EDs, only 6%–35% report adhering to evidence‐based protocols like CBT (Waller 2016), often omitting critical evidence‐based treatment elements (von Ranson et al. 2013). For example, less than 60% of self‐identified CBT therapists report using self‐monitoring, which is a core component of treatment (von Ranson et al. 2013). Digital technologies can significantly expand access to care (Fitzsimmons‐Craft, Eichen, et al. 2020) by overcoming several well‐documented barriers to ED treatment, including stigma, shame, practical barriers (e.g., cost of treatment, availability of local treatment) (Ali et al. 2017), as well as poor adherence.

Indeed, digital interventions can meaningfully improve treatment fidelity through the use of structured evidence‐based content and materials, which can increase adherence to the model (Zainal et al. 2025), and they have demonstrated effectiveness in guided self‐help formats (Linardon et al. 2017). Specifically, college women with EDs randomized to a coached digital program experienced a significantly greater reduction in ED psychopathology at post‐intervention (*d* = −0.40, *p* < 0.001) as well as through long‐term follow‐up (through 2 years; *d* = −0.35, *p* < 0.001) than those randomized to usual care. Importantly, the majority of intervention participants (83%) began the mobile intervention, whereas only 28% of control participants received ED treatment at any point during the 2‐year follow‐up period (Fitzsimmons‐Craft, Eichen, et al. 2020). Further, they require fewer resources and expertise to deliver, making them affordable and promising for lower‐income, publicly insured individuals.

While digital guided self‐help programs can improve access to care as well as treatment fidelity, standard evidence‐based interventions have not been effectively tailored to address the unique needs of individuals from lower socioeconomic status. Publicly insured or uninsured adults with EDs endorse numerous barriers to care, many of which have been highlighted in previous work (Accurso, Buckelew, et al. 2021; Accurso, Mu, et al. 2021; Regan et al. 2017), including general challenges related to insurance and finances, geographic challenges, and insufficient expertise in available providers. There are also unique treatment considerations for lower‐income individuals with EDs (e.g., impact of housing and/or food insecurity on treatment goals, including regular eating; embodying cultural humility in the discussion of food/eating; facilitating connections to additional supports, such as free or low‐cost food resources, low‐cost clothing) (Bailey‐Straebler et al. 2024). Digital interventions have a lower bar for treatment entry because they can address financial, insurance‐related, and geographic barriers. Digital self‐help programs also provide flexibility, which may increase engagement, address time‐related concerns, and preserve a sense of perceived anonymity to address concerns about negative social evaluation and shame endorsed by lower‐income individuals with EDs (Vendlinski et al. 2025). Our initial programmatic pilot testing also highlighted the need for tailored adaptation to ensure digital evidence‐based ED interventions feel inclusive, representative, and useful for individuals across social identities (e.g., age, gender, body size) and experiences (Vendlinski et al. 2025). Given health care system challenges, we leveraged technology for lower‐income, publicly insured and uninsured individuals who have relatively high digital access. Indeed, most Medicaid‐insured adults own a smartphone (86%, Deloitte Insights 2018) and have home broadband internet access (89%, SHADAC 2025). Employing a user‐centered design approach, our team adapted an existing online intervention to provide immediate, cost‐effective treatment to vulnerable individuals with EDs who have a higher likelihood of experiencing inequities in access to care.

To our knowledge, CALM‐ED (Changing Attitudes, baLance, and Mindfulness for Eating Disorders) is the first digital CBT intervention for EDs adapted for publicly insured or uninsured lower‐income adults with EDs—populations at high risk of experiencing health inequities. User‐centered design was used to guide modifications to the existing guided (coached) self‐help CBT‐based online program (Fitzsimmons‐Craft, Eichen, et al. 2020; Saekow et al. 2015) in order to be more responsive to the needs of lower‐income individuals. The study's key innovation was the development of an adapted digital intervention for lower‐income adults that employed a user‐centered design approach by engaging with stakeholders throughout the design process to ensure that the program was tailored to their specific needs, with the aim of improving engagement and clinical impact (Graham, Trockel, et al. 2019; Graham, Wildes, et al. 2019). The intervention incorporated and acknowledged critical aspects of social identity and context, enhancing its relevance and potential effectiveness (Vendlinski et al. 2025); coaching was incorporated throughout the program to increase engagement, allow for individual tailoring and personalization of strategies, and provide a level of support and accountability desired by participants. Building on our qualitative investigation, the current study evaluated the feasibility and preliminary effectiveness of the adapted intervention in an open pilot trial for publicly insured or uninsured adults with EDs who engage in frequent binge eating and/or purging behaviors.

## 1 Methods

### 1.1 Participants and Procedure

Participants were recruited through community contacts (e.g., leadership in publicly‐funded systems of care, leaders of mental health councils), social media (e.g., Twitter), and the online screener hosted by the National Eating Disorders Association (NEDA). Study advertisements indicated that publicly insured and uninsured adults with eating and body image concerns may be eligible for a three‐month coached online program at no cost that targets concerns about weight, shape, and eating habits. Individuals who endorsed ≥ 6 binge eating episodes, ≥ 6 vomiting episodes, and/or ≥ 6 laxative/diuretic episodes in the past 3 months (consistent with DSM‐5 BN, BED, or other specified feeding or eating disorder presentations (BN or BED of limited frequency) with a body mass index (BMI) of at least 18.5 kg/m^2^ were eligible. Participants were also required to be adults (18+ years) in the U.S. with public insurance or without insurance coverage who had regular access to an electronic device and internet and could read and speak English. Exclusion criteria included (1) BMI < 18.5 kg/m^2^ in the past year or a screening diagnosis of clinical or subclinical anorexia nervosa (given the potential associated medical complications and a lack of prior support for use of digital programs this population), based on a standardized screening tool (Stanford‐Washington University EDs Screen [SWED]; Graham, Trockel, et al. 2019; Graham, Wildes, et al. 2019), (2) enrollment in private health insurance and/or enrollment in Medicare with an annual income > 200% of the 2023 federal poverty level for household size (Health and Human Services Department 2023), and/or (3) current participation in a higher level of care treatment for an ED (i.e., intensive outpatient, partial hospitalization, or residential program; or inpatient hospitalization).

After providing informed consent, participants were asked to complete an online baseline assessment, after which they were given access to the coached digital intervention for 3 months. At the end of the intervention period, they were asked to complete a final online assessment. Participants were remunerated with a $35 electronic gift card at both baseline and end of treatment for completing assessments. The relevant institutional review boards approved all procedures.

### 1.2 Intervention

The intervention in this study was adapted from an existing guided self‐help CBT‐based mobile intervention for EDs that covers the core components of CBT for EDs (Fairburn and Beglin 2008) and has been previously deployed in college students with frequent binge eating and/or purging behaviors. Based on initial (Vendlinski et al. 2025) and subsequent pilot testing feedback gathered through our user‐centered design process, the program was modified to increase accessibility, acceptability, and appropriateness. User‐centered design involves assessing stakeholders' needs and adapting program content and features accordingly (Graham, Trockel, et al. 2019; Graham, Wildes, et al. 2019). First, hour‐long needs assessment interviews were conducted to understand stakeholders' experiences with ED treatment, past/current use of digital applications for mental health and/or EDs, and desires/needs for an online ED intervention (Vendlinski et al. 2025). Modifications to the intervention were made to respond to stakeholders' needs (as feasible within budgetary constraints), including features (e.g., check‐in questions at each session, interactive components, audio recordings of long text passages) and content (i.e., affordable recipes, food bank and government nutrition resources). The adapted program was subsequently shown to stakeholders in hour‐long usability testing interviews, where stakeholders provided additional feedback on program content, features, and appearance.

Content was further modified across the program (skills, infographics, worksheets, and examples) to address user feedback, which centered around increasing inclusivity and representation across diverse social identities (i.e., body size/shape, gender identity, sexual orientation, racial/ethnic identity, age, socioeconomic status, physical ability) and experiences (i.e., living in a larger body, living with chronic illness and pain, living with disability) (see Table 1 for a summary of treatment considerations and adaptations made to the program through the user‐centered design approach). In addition, the program design was adapted with audio recordings to accompany every text passage, additional graphics, and simplified text passages in response to feedback about diversity in physical ability and the need for increased accessibility. Further, the program was adapted in response to feedback about how the application of skills and/or content may need to be significantly adapted to suit individuals' unique circumstances and structural barriers (e.g., regular eating in the context of food insecurity). Language was incorporated across sessions to acknowledge the role of structural, systemic, and institutional inequities that impact ED care. Finally, the program was adapted to provide clearer framing around intervention style and structure, goals, and realistic expectations for the program. In line with these adaptations, the coaching component of the program was designed to consistently support and empower users to individualize the use of the intervention based on their personal goals and unique circumstances.

**TABLE 1:** Identified ED treatment barriers, considerations, and adaptations.

Barriers	Adaptations
Financial and geographic access barriers	Access to CALM‐ED was provided to participants free of cost, and for 6 months after the study ended. Program can be accessed online via smartphone or computer.
Structural barriers (e.g., inflexible work schedules, multiple jobs, caretaking responsibilities)	Participants were encouraged to engage in the program flexibly to increase their sense of agency. Adapted coaching to support participants in individualizing use of the program to fit their unique circumstances.
Lack of individualized care	Adapted content to increase representation across social identities and intersections (i.e., body shape/size, gender identity, sexual orientation, racial/ethnic identity, age, socioeconomic status, and physical ability). Adapted content and coaching to acknowledge and address individual lived experiences and circumstances that may impact low‐income individuals (e.g., unstable housing, food insecurity, chronic health conditions, disability). Adapted content and coaching to encourage participants to engage in the intervention flexibly and with respect to their individual goals and needs. Adapted content to acknowledge and validate structural, systemic, and institutional inequities that can impact ED care.
Lack of social support	Adapted coaching to both facilitate engagement in the program and provide support for participants.
Social stigma and shame	Coaching was provided via text messaging to protect participant privacy and anonymity. Adapted coaching to normalize and validate participant experiences, encourage self‐compassion, and provide psychoeducation on EDs to decrease stigma and shame. Adapted content (i.e., vignettes, examples) to increase representation of individuals across diverse identities.
Accessibility barriers	Adapted intervention to increase content accessibility (i.e., audio recordings, informational graphics, and simplified text passages).

The program covers the core components of CBT for EDs (Fairburn and Beglin 2008) with key targets (i.e., decreased dietary restraint and weight/shape concerns) addressed early in the program to maximize efficiency. The program includes self‐monitoring, goal setting, meal planning and tracking, and cognitive‐behavioral strategies to improve body image and eating. Other content addresses media wellness, relationship and communication issues, and relapse prevention. To minimize costs, the program was delivered via the Qualtrics platform, which was available at no cost to the research team and could be configured to deliver program content for this initial pilot and feasibility trial. Participants had flexible pacing options to enhance their agency in using the program (Nitsch et al. 2016), which facilitates consumer‐driven delivery and is associated with improved engagement (Donkin and Glozier 2012). Specifically, participants could engage with sessions in short bursts of time, in their preferred order, rather than requiring sessions to be completed sequentially. In this guided self‐help intervention, coaches were available to support and enhance user motivation, monitor progress, facilitate goal setting, offer accountability, provide feedback on technique usage, encourage practice, answer user questions, and monitor/manage clinical risk. Coaching involved twice weekly communication with users through SMS to provide feedback, check in on progress, and reinforce program use and efforts over the 3‐month intervention period. Coaching was delivered by two pre/postdoctoral clinical psychology fellows with specialized training in EDs (KYY, CRD) under weekly group supervision by licensed psychologists (EFC, ECA). Given the study's preliminary nature, a three‐month time frame was optimal to study the feasibility and acceptability of the intervention for this population, and similar to the amount of time offered across many other digital guided self‐help interventions for EDs (8–12 weeks: Aardoom et al. 2016; Melisse et al. 2023; Jenkins et al. 2021; Hildebrandt et al. 2017).

### 1.3 Measures

At baseline, participants provided demographics, past year health insurance coverage, average daily time spent on a smartphone, age of ED onset, past year intent to seek ED treatment, barriers to accessing ED care (used in Fitzsimmons‐Craft, Eichen, et al. 2020, which were modified from Cachelin and Striegel‐Moore 2006), current and prior ED treatment (including engagement in any online treatment, online resources, or apps), and food insecurity risk (based on the Hunger Vital Sign questionnaire). Feasibility and acceptability were assessed by documenting participant recruitment rates (i.e., percent of eligible individuals who enrolled in the study), adoption (i.e., percent of enrolled individuals who initiated program use through logging in at least once), intervention engagement (e.g., number of sessions completed, total number of minutes spent engaging in online program, number of times sessions were revisited, number of text messages/total words to coach, number of text messages/total words from coach), and study retention rates (i.e., percent follow‐up completion), and end‐of‐treatment usability and acceptability. Preliminary effectiveness was also assessed through the collection of primary outcomes (ED psychopathology, objective binge episodes, compensatory behavior episodes, and ED‐related clinical impairment) and secondary outcomes (depression, anxiety) at the end of treatment.

Mobile App Rating Scale (MARS) (Stoyanov et al. 2015). This scale assesses the quality of health apps. In the current study, we used two subjective quality and six app‐specific questions from the MARS as descriptive measures to assess the perceived quality and impact of the CALM‐ED program.

System Usability Scale (SUS) (Sauro 2011). This scale measures perceived usability of any given information system and consists of 10 items rated on a 5‐point Likert scale, ranging from strongly disagree (1) to strongly agree (5). Scores are scaled from 0 to 100, with a SUS score over 68 delineating “above average” usability. Cronbach's alpha in the current study was 0.91.

Eating Disorder Examination‐Questionnaire (EDE‐Q) (Fairburn and Beglin 2008). This 28‐item measure assesses the overall severity of ED psychopathology and the frequency of ED behaviors in the past month (e.g., objective binge eating, self‐induced vomiting, laxative misuse, and compulsive/driven exercise). Higher global scores reflect more severe eating‐related difficulties. In this study, Cronbach's alpha for the EDE‐Q global score ranged from 0.85 at baseline to 0.90 at follow‐up.

Clinical Impairment Assessment (CIA) (Bohn et al. 2008). This 16‐item measure of impairment assesses how eating behaviors, exercise behaviors, or eating/body‐related cognitions affected functioning over the past four weeks. Responses were rated on a four‐point Likert scale, where 0 indicates “not at all” and 3 represents “a lot.” Cronbach's alpha ranged from 0.90 to 0.92 across timepoints.

Patient Health Questionnaire‐9 (PHQ‐9) (Kroenke and Spitzer 2002). This 9‐item scale assesses major depressive disorder, with each item rated on a four‐point Likert scale (0–3). Total scores range from 0 to 27, with higher scores indicating greater severity of depression. Scores over 10 indicate moderate to severe depressive symptoms. In the current study, Cronbach's alpha for the PHQ‐9 was 0.82 at baseline and 0.87 at follow‐up.

Generalized Anxiety Disorder‐7 (GAD‐7) (Spitzer et al. 2006). This 7‐item measure assesses the symptoms of generalized anxiety disorders. Each item is rated on a four‐point Likert scale (0–3), with total scores ranging from 0 to 12. Scores of 10 or greater are considered to be in the clinical range. Cronbach's alpha ranged from 0.89 to 0.92 across time points.

### 1.4 Analysis

Descriptive statistics describe primary outcomes (i.e., participant enrollment and treatment engagement), with ED psychopathology as a secondary clinical effectiveness outcome. Pilot effect sizes were calculated by comparing baseline to 3‐month follow‐up scores using paired samples *t* tests and Cohen's *d*. Using the method outlined by Jacobson and Truax (1991), taking the difference of the standard errors, we also calculated a reliable change index (RCI) across clinical outcome measures to determine the percentage of participants who reported a reliable improvement in symptoms from pre‐ to post‐intervention, excluding objective binge or compensatory behavior episodes since these violated the assumption of a normal distribution. Based on this approach, we considered a reliable improvement to be met if the reduction from pre‐ to post‐intervention score was greater than the RCI for each outcome: 0.42 (EDE‐Q global), 3.90 (CIA), 2.41 (PHQ‐9), and 2.30 (GAD‐7).

## 2 Results

**FIGURE 1:**
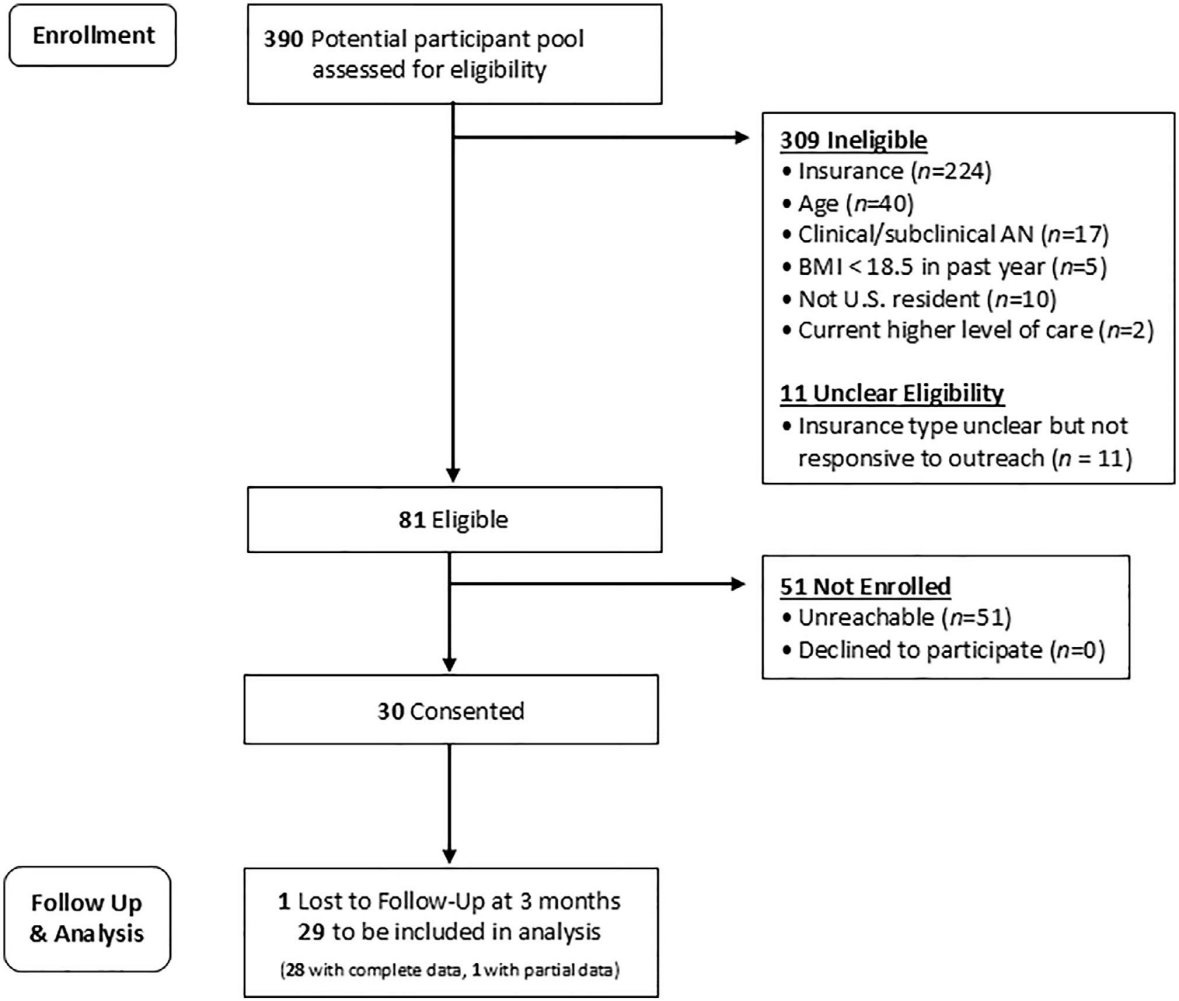
Consort flow diagram.

Of the 81 eligible potential participants, 30 (37.0%) were responsive to initial outreach, all of whom consented to participate (see Figure 1 for the consort flow diagram). At entry, most participants (90%, *n* = 27) screened positive for bulimia nervosa or subthreshold bulimia nervosa (see Table 2). The severity of ED symptomatology was found to be high, with a 20‐year average duration of illness and very frequent binge eating episodes and inappropriate compensatory behaviors in the prior 3 months (i.e., a combined total average of 8.04 ED behaviors per week). Even among participants with subthreshold bulimia nervosa, 80% reported either engaging in binge eating (*n* = 1, 40 episodes over 3 months) or compensatory behaviors (*n* = 7, M = 45.6 episodes over 3 months) greater than once per week. All participants met the 2023 federal poverty level based on household size (Health and Human Services Department 2023); two‐thirds (*n* = 20) of participants had a yearly household income of $21,960 or less. Demographic characteristics indicated several psychosocial stressors (e.g., more than half were at high risk for food insecurity). Nearly two‐thirds (63.3%, *n* = 19) held at least one minoritized identity, including sexual and/or gender minorities (36.7%, *n* = 11) and individuals from minoritized racial and/or ethnic identities (36.7%, *n* = 11).

**TABLE 2:** Demographic and clinical characteristics of participants.

	Mean (SD) or *n* (%)	Range	Mdn
Age	34.17 (13.29)	19–64	30.5
Duration of illness (yrs)	20.60 (14.86)	1–54	17.5
Gender identity			
Female	22 (73.3%)		
Male	3 (10.0%)		
Transgender male	2 (6.7%)		
Non‐binary	3 (10.0%)		
Hispanic/Latinx ethnicity	4 (13.3%)		
Race^a^			
American Indian or Alaska Native	3 (10.0%)		
Asian	2 (6.7%)		
Black or African American	3 (10.0%)		
White	20 (66.7%)		
Other	2 (6.7%)		
LGBTQ+	11 (36.7%)		
Living with a physical disability	7 (23.3%)		
Caregiver for someone with a physical/mental health illness	6 (20.0%)		
High risk for food insecurity	16 (53.3%)		
Health insurance coverage			
Medicaid	17 (56.7%)		
Medicare	2 (6.7%)		
Other qualified health plan under CMS	2 (6.7%)		
None	9 (30.0%)		
Screening diagnosis			
Bulimia nervosa	17 (56.7%)		
Subthreshold bulimia nervosa	10 (33.3%)		
Binge eating disorder	2 (6.7%)		
Subthreshold binge eating disorder	1 (3.3%)		
Eating disorder behaviors over the past 3 months	96.53 (71.23)	10–254	70
Objective binge episodes	35.87 (30.78)	1–100	30
Self‐induced vomiting	11.87 (23.35)	0–80	0
Other inappropriate compensatory behaviors	48.80 (47.47)	0–170	31
Body mass index	33.73 (10.21)	22.04–60.26	31.52

*Note: N* = 30. “Other inappropriate compensatory behaviors” included frequency of laxative misuse, driven compulsive exercise, fasting, and diet pill use.

Abbreviations: CMS, Centers for Medicare & Medicaid Services; Mdn, Median.

^a^ Race and Other minoritized groups percentages add up to > 100% due to some participants selecting multiple categories.

### 2.1 Treatment History

Half of the participants (50.0%, *n* = 15) had sought ED treatment in the past year, but less than a quarter (23.3%, *n* = 7) had received it. Five participants reported actively receiving therapy (16.7%, *n* = 5), but only two indicated that eating‐related concerns were frequently addressed in their current treatment. Additionally, about two‐fifths had ever received any ED treatment (43.3%, *n* = 13). In addition to formal treatment, 60% (*n* = 18) were currently or had previously engaged in online ED support (e.g., ED app use, virtual ED support groups, support through social media). Of the participants who neither sought nor received ED treatment in the past year (46.7%, *n* = 14), common barriers to accessing care included believing that one should be able to manage these problems independently and not thinking the problem was severe enough to warrant treatment, despite very low endorsement of not thinking that one had problems with eating and/or weight (Table 3).

**TABLE 3:** Barriers to seeking and receiving treatment.

Barrier	M	SD	% endorsed
1. I felt shame or embarrassment	3.29	1.38	50.0
2. I have not known where to go to find help	3.57	1.45	64.3
3. I believed I should be able to help myself	4.64	0.74	85.7
4. I worried about being labeled or judged	3.50	1.61	64.3
5. I believed that my problem was not serious enough to warrant treatment	3.93	1.64	78.6
6. Treatment was too expensive	3.57	1.50	50.0
7. I have been unaware of the different treatment options available	3.79	1.37	64.3
8. I have had a lack of trust in providers	3.07	1.27	50.0
9. I have turned to other sources of support such as family and friends	2.29	1.20	21.4
10. I have had a lack of social support for seeking treatment	3.21	1.58	50.0
11. I worried that providers would lack expertise or not understand my issues	3.00	1.36	42.9
12. I have not believed that an eating disorder is a psychological problem	2.36	1.45	35.7
13. I have not had transportation to get to treatment	1.57	1.09	14.3
14. I have been concerned about stigma	2.50	1.34	35.7
15. I did not have time to seek treatment	3.29	1.49	57.1
16. I do not believe that I have any problems with my eating or weight	1.57	0.76	0.0
17. Providers or treatment centers did not take my insurance	2.43	1.34	14.3
18. I did not have insurance to cover my treatment	3.00	1.66	42.9

*Note: N* = 14 participants who reported not seeking or receiving any treatment for eating problems in the past year. All items were rated on a 1 (*strongly disagree*) to 5 (*strongly agree*) scale. Individuals who rated an item as a 4 (agree) or 5 (strongly agree) comprised those participants represented in % endorsed.

### 2.2 Engagement

Almost all participants started (93.3%, *n* = 28) and completed (83.3%, *n* = 25) the introductory session. Most participants (61.5%, *n* = 16) started at least half of the eight core sessions (see Table 4), and about one quarter (23.1%, *n* = 6) started all sessions. Over the 3‐month treatment period, participants (*n* = 26) who accessed the program completed an average of 4.15 sessions (SD = 2.68) and spent an average of 8.25 h (SD = 9.34) engaging with online program content. On average, participants engaged with each session 2–3 times to complete the session in multiple sittings and revisit prior content. Per participant, coaches sent an average of 47.6 text messages (SD = 26.6) containing 2068.8 words (SD = 1099.6). All but one participant responded to coaching texts (96.7%, *n* = 29). Participant responsiveness was highly variable but indicated significant engagement overall (total text messages to coach: median = 27, M = 32.5, SD = 35.2, range: 0–184; total SMS words to coach: median = 401.5, M = 953.5, SD = 1207.2, range: 0–4940).

**TABLE 4:** Program metrics for engaged participants.

	M	SD	Range		
Total time spent in program (h)	8.25	9.34	0.58–33.52		
Number of sessions started (out of 8)	4.15	2.68	1–8		
Number of sessions completed (out of 8)	3.29	2.54	1–8		

	Participants started		Number of times visited		
	*n*	%	M	SD	Range
Session 1	25	96.2	3.24	2.55	1–9
Session 2	19	73.1	2.21	1.18	1–5
Session 3	17	65.4	2.06	1.39	1–5
Session 4	13	50.0	2.23	2.24	1–9
Session 5	10	38.5	2.70	3.53	1–12
Session 6	10	38.5	3.00	3.40	1–12
Session 7	7	26.9	2.71	2.93	1–9
Session 8	7	26.9	2.14	0.90	1–3

*Note: N* = 26.

### 2.3 Acceptability and Usability

The CALM‐ED program demonstrated good acceptability overall (see Table 5). On average, coaches were rated as very helpful and caring. Participants rated the program as likely to increase awareness and knowledge, increase access to information about eating and body image concerns, and change attitudes and behaviors related to these issues (all average scores > 3.50 on a 6‐point scale). In addition, two‐thirds (*n* = 18) anticipated continuing to access the program at least occasionally over the next nine months. Usability based on the SUS (M = 62.40 out of 100, SD = 25.78, range: 0–97.50) was about one‐half standard deviation below the average (across 206 studies: M = 69.69, mean of SDs = 18.00; Bangor et al. 2008).

**TABLE 5:** Program acceptability and usability results.

	M	SD
**Satisfaction with coach and program**		
How helpful was your coach with your goals for the program?	5.11	1.20
How satisfied are you with the feedback your coach provided?	4.93	1.18
How caring was your coach toward you?	5.39	1.10
How relevant were your coach's suggestions to the issues that you brought up?	4.89	1.29
How helpful was the CALM‐ED program with your eating and body image concerns?	4.07	1.65
How helpful was CALM‐ED compared to other treatments you have received for your eating and body image concerns (if applicable)?	4.36	1.55
How relevant were the CALM‐ED program modules to the issues you experience with eating and body image concerns?	4.75	1.11
**MARS program‐specific questions**		
Awareness: This program is likely to increase awareness of the importance of addressing eating and body image concerns	4.04	1.11
Knowledge: This program is likely to increase knowledge/understanding of eating and body image concerns	4.23	1.03
Attitudes: This program is likely to change attitudes toward improving eating and body image concerns	3.96	1.11
Intention to change: This program is likely to increase intentions/motivation to address eating and body image concerns	3.69	1.19
Help seeking: Use of this program is likely to encourage further help seeking for eating and body image concerns	4.08	1.13
Behavior change: Use of this program is likely to decrease eating and body image concerns	3.81	1.10
**SUS score**	62.40	25.78

*Note: N* = 28. Individual items about satisfaction and program‐specific questions were rated on a 1 to 6 scale.

Abbreviations: MARS, Mobile Application Rating Scale; SUS, System Usability Scale.

### 2.4 Satisfaction

Most (67.9%, *n* = 19) participants indicated that they were somewhat to very satisfied with the program, and half (*n* = 14) had gained at least some of what they wanted from the program.

### 2.5 Effectiveness Outcomes

From pre‐ to post‐intervention, there were statistically significant and large reductions in the EDE‐Q global score (*d* = 0.79, *p* < 0.001) and moderate reductions in binge eating (*d* = 0.62, *p* = 0.003) and compensatory behavior episodes (*d* = 0.49, *p* = 0.016) (see Table 6). Clinical impairment, depression, and anxiety were all significantly improved as well, with medium to large effects. Over half of the sample demonstrated reliable improvements in ED psychopathology and ED‐related clinical impairment. Reliable improvements in depression and anxiety were demonstrated by 61% and 43% of the sample, respectively.

**TABLE 6:** Paired samples *t*‐test results for pre‐post survey.

		Baseline		3‐month follow‐up					Reliable improvement % (*n*)
Measure	*n*	M (SD)	Range	M (SD)	Range	*t*	*d*	*p*	
Global EDE‐Q score	29	4.21 (1.25)	0.28–5.65	3.30 (1.27)	0.00–5.10	4.27	0.79	< 0.001	51.7% (*n* = 15)
Objective binge episodes	28	16.39 (19.44)	0–100	6.46 (5.70)	0–25	3.27	0.62	0.003	—
Compensatory behaviors	28	17.87 (19.61)	0–84	12.93 (20.87)	0–102	2.58	0.49	0.016	—
CIA	28	39.93 (9.74)	1.00–47.00	24.21 (10.71)	0.00–44.00	4.39	0.83	< 0.001	57.1% (*n* = 16)
PHQ‐9	28	17.07 (5.41)	6.00–27.00	11.64 (6.52)	1.00–27.00	4.42	0.84	< 0.001	60.7% (*n* = 17)
GAD‐7	28	14.64 (5.63)	2.00–21.00	11.71 (6.87)	0.00–21.00	2.5	0.47	0.019	42.9% (*n* = 12)

*Note:* Objective binge episodes and compensatory behaviors (including totals for self‐induced vomiting, laxative use, and compulsive exercise) were assessed via the EDE‐Q (i.e., total number of times in the past 28 days).

Abbreviations: CIA, Clinical Impairment Assessment; EDE‐Q, Eating Disorder Examination‐Questionnaire; GAD‐7, Generalized Anxiety Disorder‐7; OBE, objective binge episode; PHQ‐9, Patient Health Questionnaire‐9.

## 3 Discussion

The primary aim of this study was to examine the feasibility and acceptability of a guided self‐help online treatment program for binge eating and purging behaviors for lower‐income publicly insured or uninsured adults with EDs. Participants in this sample represent an underserved population at high risk for health disparities. Despite long‐standing and relatively severe EDs (primarily bulimia nervosa), most participants were naïve to ED treatment. The level of engagement in this intervention appeared relatively high with respect to treatment initiation (93%), sessions started (52%—4.2 of 8 sessions), and sessions completed (41%—3.3 of 8 sessions). Participants also revisited content frequently (more than eight hours of online program engagement, on average) and used coaching several times per week.

Engagement was comparable to other digital interventions for adults with ED symptoms that employed similar asynchronous text‐based coaching support across treatment initiation (83%: Fitzsimmons‐Craft, Taylor, et al. 2020), content started (68%: Saekow et al. 2015), and sessions completed (31%: Fitzsimmons‐Craft, Taylor, et al. 2020). As expected, our treatment completion rates were comparable or lower when compared to digital interventions that included live therapy sessions (45%: Puls et al. 2020; 84%: Rom et al. 2023) given higher rates of engagement in traditional face‐to‐face delivery than internet‐based CBT (Linardon et al. 2018). Nevertheless, the findings from this study are promising, with high levels of satisfaction with both the online program and coaching, suggesting excellent feasibility and acceptability. Our preliminary investigation of effectiveness was also encouraging, with large improvements in ED psychopathology and clinical impairment and moderate decreases in binge eating and compensatory behaviors at the end of treatment. Compared with other uncontrolled trials that offered guided self‐help including eight 30‐min live coaching sessions, this study demonstrated greater rates of reliable improvement than one trial (ED psychopathology: 52% vs. 38%: Vaz et al. 2013) but with smaller effects than the other trial (ED psychopathology: *d* = 0.79 vs. 1.59; binge eating episodes: *d* = 0.62 vs. 0.89; Dalton et al. 2024). These results are preliminary and should be interpreted with caution given the study's small sample size and uncontrolled design.

This study has several strengths, including its focus on lower‐income, publicly insured and uninsured adults with EDs, who have traditionally been underserved in usual care settings. Evidence‐based, cost‐effective, easy to disseminate interventions are critically needed, given that specialized services for EDs are lacking in publicly funded health care systems (Accurso et al. 2025). In addition, the existing evidence‐based intervention (initially developed for female college students) was adapted to incorporate perspectives across social identities. While most participants were White, nearly two‐thirds of the sample held at least one minoritized identity, including approximately one‐third of participants of color and another third of participants who were sexual and/or gender minorities. Further, this study was informed by a user‐centered design approach that incorporated mixed methods to address the needs of lower‐income adults with EDs while maintaining evidence‐based intervention elements. However, this pilot study had several important limitations, including its use of an uncontrolled design with a small English‐speaking sample, short intervention timeframe, and short assessment timeframe with clinical outcomes measured at the end of the pilot intervention to detect a signal (i.e., early change as a strong predictor of longer‐term outcome). While the platform usability ratings were slightly below average, this was to be expected given our decision to employ a survey platform instead of one designed specifically for delivering online programs. As a result, there were notable costs to the user experience (e.g., aesthetics, flexibility, ease of navigation). With preliminary data supporting its effectiveness, future intervention iterations can invest in platforms specifically designed for online program delivery to improve usability.

This is the first digital intervention specifically tailored for lower‐income adults with EDs featuring frequent binge eating and/or purging. The results from this pilot trial testing a brief online guided self‐help intervention are promising, with relatively high treatment engagement, indicating good feasibility and acceptability and a preliminary signal of potential effectiveness. Future research can leverage this adapted intervention to examine its medium‐ (6–9 months) and longer‐term (2‐year) impact relative to other active treatments or waitlist control in a randomized design.

## Author Contributions

**Erin C. Accurso:** conceptualization, data curation, formal analysis, funding acquisition, investigation, methodology, resources, software, supervision, writing – original draft, writing – review and editing. **Catherine R. Drury:** data curation, formal analysis, writing – original draft, writing – review and editing. **Kimberly Yu:** data curation, investigation, writing – original draft, writing – review and editing. **Siena Vendlinski:** data curation, methodology, project administration, writing – review and editing. **Nancy Jacquelyn Pérez‐Flores:** writing – original draft, writing – review and editing. **Carli P. Howe:** project administration, writing – review and editing. **Denise E. Wilfley:** conceptualization, writing – review and editing. **Ellen E. Fitzsimmons‐Craft:** conceptualization, data curation, funding acquisition, investigation, methodology, resources, supervision, writing – original draft, writing – review and editing.

## Conflicts of Interest

Dr. Accurso has consulted with Partnership HealthPlan of California (a healthcare organization that contracts with the state to administer Medicaid benefits) concerning strategies to improve the treatment of eating disorders. The other authors have no conflicts to declare.

## Data Availability

The data that support the findings of this study are available from the corresponding authors upon reasonable request.
